# Perioperative bridging anticoagulation for atrial fibrillation—the first randomised controlled trial

**DOI:** 10.1186/s13741-016-0040-5

**Published:** 2016-06-07

**Authors:** James Matthew Wight, Malachy Oliver Columb

**Affiliations:** Department of Anaesthesia and Perioperative Medicine, University College Hospital, London, UK; Department of Anaesthesia and Intensive Care Medicine, University Hospital of South Manchester, Wythenshawe, UK

**Keywords:** Anticoagulation, Atrial fibrillation, Bleeding, Bridging, Low molecular weight heparin, Thromboembolism, Warfarin

## Abstract

**Abstract:**

Patients who have atrial fibrillation (AF) have increased thromboembolic risk. This risk is mitigated through use of anticoagulants, traditionally with vitamin K antagonists such as warfarin, and more recently with drugs such as Xa and thrombin inhibitors. Since anticoagulants increase the risk of bleeding, uncertainty exists regarding their use in the perioperative period. The risk of thromboembolism for each patient must be balanced against risk of bleeding; anticoagulation medication may be continued, replaced with a short-acting alternative or withheld entirely. Until recently, evidence on best management relied on expert opinion and observational studies. The recent publication of a randomised, double-blind, placebo-controlled trial (BRIDGE) has added important information to the knowledge base.

**Trial registration:**

BRIDGE ClinicalTrials.gov, NCT00786474

## Background

Every year, approximately 10 % of warfarinised patients with AF present for surgery or invasive procedure (Douketis et al. 2012; Healey et al. 2012). Clinicians must decide which patients require interruption of their anticoagulation therapy, timing of any interruption and whether to substitute a usual anticoagulant with a short-acting alternative. Reviews and guidelines published to support this decision-making process have been based on expert opinion and observational studies, and calls for further research and high quality trials have been made by many (Douketis et al. 2012; Wysokinski et al. 2008; Garcia et al. 2008; Eckman 2005; Ickx and Steib 2006).

## Main text

### New research

Douketis et al. present the first randomised controlled trial of the benefits and risks of bridging anticoagulation (Douketis et al. 2015). Patients (*N* = 1884) with AF had their warfarin stopped 5 days before surgery and received either twice daily dalteparin (100 units kg^−1^) or placebo from 3 days until 1 day before surgery. Warfarin was restarted on the day of or the day after surgery. Dalteparin or placebo was restarted at 12–24 or 48–72 h after surgery for low or high bleeding risk procedures respectively and was continued until the INR was 2.0 or higher.

The primary efficacy outcome was the incidence of arterial thromboembolism (ATE) at 30 days (stroke, systemic embolism, transient ischaemic attack), and the primary safety outcome was major bleeding at 30 days. The authors performed a one-sided ‘non-inferiority’ test within a margin of 1 % of placebo to dalteparin comparing ATE rates. A two-sided ‘superiority’ test was performed to compare major haemorrhage rates. The study had 90 % power for the two primary end points.

### Results

The incidences of ATE were 0.3 % with dalteparin and 0.4 % with placebo; placebo was non-inferior to dalteparin (*P* < 0.01). The incidences of major haemorrhage were 3.2 % with dalteparin and 1.3 % with placebo; placebo was superior to dalteparin (*P* < 0.005).

### Critique

Perioperative staff commonly face uncertainty as to the best management strategy for patients, presenting for surgery, who are taking warfarin for AF. This study addresses an important and common issue that has previously lacked high-quality research.

Some issues with the study design and conclusions have been raised (Duca et al. 2016). On closer examination of the study population, less than 3 % of patients had the highest CHADS_2_ scores of 5 or 6. These patients have the highest annualised stroke risk (at 12–18 %) and would potentially benefit most from perioperative bridging anticoagulation (Gage et al. 2001). In addition, many surgical procedures associated with high rates of ATE were not represented at all, including carotid endarterectomy and major cancer surgery. Concluding that omission of bridging anticoagulation is non-inferior to use of bridging anticoagulation for all may cause disadvantage to these high-risk groups.

The diagnosis of ATE within the protocol required development and recognition of clinical symptoms. Silent ATE occurs more commonly than overt ATE and is associated with long-term complications (Sacco et al. 2013; Fanning et al. 2014). The results may therefore not reflect the true impact of omitting bridging anticoagulation.

The vast majority of procedures performed were associated with a low risk of bleeding, and almost 40 % of patients underwent minor gastrointestinal procedures including endoscopy. Preexisting guidelines state that such procedures may be performed without interruption of oral anticoagulation (Veitch et al. 2008). Without further information on the exact procedures carried out, and without subgroup analysis, it is difficult to know if the major conclusions apply to particular patient populations.

The study protocol randomised patients to receive high-dose dalteparin or placebo. In practice, if warfarin was withheld and high-dose dalteparin was not to be given, many hospital guidelines would advocate administration of lower dose low molecular weight heparin for venous thromboembolism prophylaxis (www.guysandstthomas.nhs.uk/resources/our-services/acute-medicine-gi-surgery/elderly-care/periop-warfarin-adults.pdf) Accessed 2 Feb 2016). How the results of the BRIDGE study would affect these recommendations is unclear. Also, this study did not investigate newer anticoagulants, which are increasingly popular and have shorter half-lives (Baron et al. 2013). The ability to continue these agents closer to the time of surgery may reduce or eliminate the requirement for bridging anticoagulation therapy.

The authors understandably tested at a 1 % margin for a one-sided hypothesis of non-inferiority of placebo to dalteparin with regard to the incidence of ATE. However, their use of the term ‘superiority’ for the two-sided hypothesis for haemorrhage is a common misuse of the term, as ‘superiority’ is also a one-sided hypothesis. The two-sided hypothesis is simply the standard ‘inequality’ hypothesis when the usual null hypothesis is rejected (Columb and Lutz 2009). Another, not uncommon problem in RCTs that the authors had to deal with was the lower than expected event rates for ATE. However, an advantage with non-inferiority designs is that the study remained adequately powered as the non-inferiority margin of 1 % became proportionately larger with respect to the lower incidence rates.

## Conclusion

This is the first reported large, multicentre, randomised, double-blind, placebo-controlled trial into perioperative bridging anticoagulation. Its findings that bridging anticoagulation may be of no benefit in preventing ATE and may increase incidence of bleeding are consistent with earlier observational studies. Although some methodological issues are present, its overall conclusions are important and highly relevant to the perioperative physician.

## Abbreviations

AF, atrial fibrillation; ATE, arterial thromboembolism
